# Scholarly publishing threatened?

**DOI:** 10.1080/03009734.2016.1238426

**Published:** 2016-10-18

**Authors:** Arne Andersson, Joey Lau Börjesson

How many invitations did you receive last week to publish your latest research data in a journal with a name very similar to those of our classic scientific journals? The reason for this generosity must be the high chance of cheating researchers so that they pay article processing charges. These journals have been denominated ‘predatory journals’ and have interested a Canadian librarian, Charles Beall, to a very high extent. The so-called ‘Beall’s list of predatory journals’ has become widely recognized (1), and today it contains more than 1000 titles. Since the number of ‘invitations’ seems to increase constantly there must be some authors that are accepting these offers. This might also be one reason for why traditional scientific journals have been facing decreasing numbers of submissions for a while. So when such offers appear in your inbox, do consult this Beall’s list. If indeed, which most often is the case, you find the journal on that list, you should forget about submitting anything to them. There is a great risk that besides losing some money you will also lose control of your manuscript. It might just disappear in cyber space or be blocked/lost in a production process that goes on forever.

One more threat to scholarly publishing is that of the shortcomings of the peer review process. That aspect was the subject of a debate article a couple of years ago by Hanna Norsted in a neighbourhood journal (2). The title of her paper was ‘The Death of the Scientific Journal’, and it focused on the fate of a spoof article fabricated by John Bohannon of *Science*. It was submitted to 304 open-access journal, and interestingly more than half of the journals accepted the article for publication. To what extent this flaw of the peer review process was caused by peer review fraud or not was not looked at. Perhaps that was because the phenomenon has become so much highlighted lately by all the retractions that had been found to be published after peer review fraud (3). This was first reported four years ago when an East Asian researcher admitted that he had invented e-mail addresses so that the review requests all landed in the inbox of himself or his friends. By such means he produced faked reviews that were submitted to the editor very quickly, and they were always in favour of acceptance of the manuscript. After that other reports on similar deceptions have appeared. The reason for the success with this sort of hacking of the review process has been that authors have been given the opportunity to recommend referees for their papers. Editors have always and perhaps more so these days had difficulties in finding reviewers that are willing to do the job. Therefore, it has seemed like a good idea to use this practice. More and more these opportunities seem to disappear. Thus, our publisher, Taylor & Francis, has removed the possibility in the manuscript central for authors to influence the identity of the reviewer. Still, authors can name persons whom they oppose to see as reviewers of their papers.

**Figure 1.**
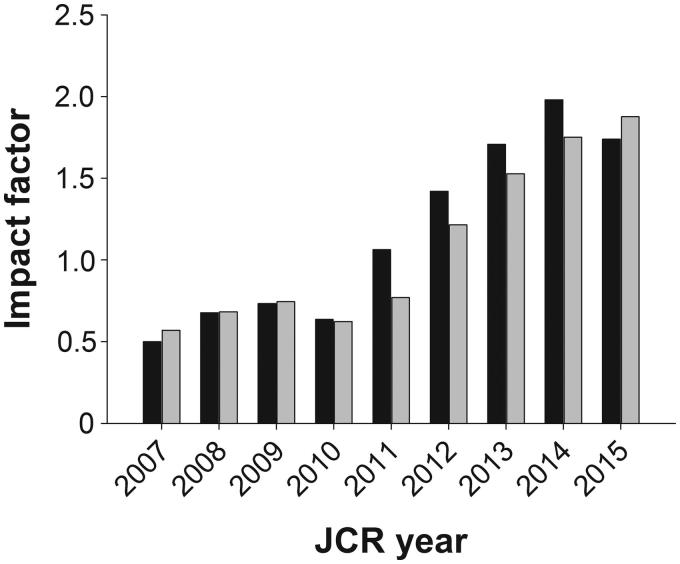
Impact factor of *Upsala Journal of Medical Sciences*. Black bar represents impact factor per JCR year and grey bar represents the 5-year impact factor.

**Figure 2.**
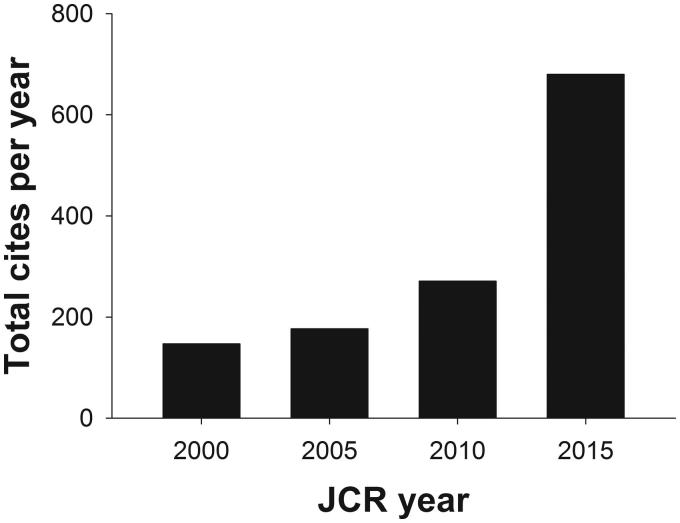
Total cites of *Upsala Journal of Medical Sciences*.

Anyhow, our journal has survived for more than 150 years in spite of both technical changes and some periods of falling interest. The 150-year jubilee was celebrated with the publication of a special issue (4,5) and an afternoon seminar at the Medical History Museum in Uppsala. In that special issue we reported on both past and future challenges, the latter in light of ‘the era of impact factor mania’ we are operating in (6). After five years of annual increases of the impact factor we have now seen a slight drop in the 2015 value (Figure 1). But at the same time the five-year impact factor—indeed many people regard this value as more relevant as it is less dependent on the impact of single articles—went on increasing and is now approaching the critical 2.0 level. Another token of our increased impact on scholarly publishing is the development of the number of total cites of *UJMS* papers (Figure 2). With some 200 yearly cites before installing the electronic manuscript central in 2009 and making all our previously published papers available for everyone on our web site we have increased the number of cites by a factor of three.

While impact factor figures tell us about the citation fate of an average article of the journal during a certain time period, it might also be of interest to see how often individual papers become very frequently cited. Or in other words, with what frequency does it happen that a certain journal publishes a scientific blockbuster? In a bibliometric publication from the Swedish Research Council some years ago Swedish scholarly publishing was compared with that in other European countries (7). Figures were then given for how many times a paper had to be cited during the two first years plus the publication year in order to become classified as the ‘more than 90th (more than 7 times) percentile’ or ‘more than 99th (more than 21 times) percentile’ cited publications. By such means it was possible to find out if a paper belonged to the 10% or 1% most cited papers for a specific publication year. To our knowledge there are no such figures calculated for a wide range of journals. We have, of course, calculated them for *UJMS*, utilizing the Thomson Reuter database. For the period 2010–2014 we have published 227 papers, and we have had three papers qualifying for the 1% and 23 for the 10% category. Thus, we can claim that we, in this respect, behave like the average journal in the Thomson Reuter database. Two of the top-cited papers, Nazarenko et al. (8) and Nordkvist and Oreland (9), were invited papers, thus supporting the idea that invited reviews tend to become the most cited papers (6). The third, by Rong et al. (10), is an original article from China reporting on the expression of miR-146a in hepatocellular carcinomas.

Although the threats towards our journal seem fairly weak, we consider the slight decrease of the submission rate as a problematic phenomenon. Against that background the enthusiasm from the board of the Upsala Medical Society for starting the use of article processing charges was meagre at our last meeting. No doubt, the combination of full open-access publishing without charging the authors any sort of fees is not that common, and it implies that people, i.e. editors, editorial board members, and referees, are willing to put a lot of their free time into the project still keeping the quality flag high and waving.
